# Eleven secondary cancers after hematopoietic stem cell transplantation using a total body irradiation-based regimen in 370 consecutive pediatric and adult patients

**DOI:** 10.1186/2193-1801-2-424

**Published:** 2013-08-30

**Authors:** Mami Omori, Hideomi Yamashita, Akihito Shinohara, Mineo Kurokawa, Jyunko Takita, Mitsuteru Hiwatari, Keiichi Nakagawa

**Affiliations:** Departments of Radiology, University of Tokyo Hospital, 7-3-1, Hongo, Bunkyo-ku, 113-8655 Tokyo, Japan; Hematology and Oncology, University of Tokyo Hospital, Tokyo, Japan; Pediatrics, Graduate School of Medicine, University of Tokyo Hospital, Tokyo, Japan

**Keywords:** Total body irradiation, Secondary cancer, Bone marrow transplantation

## Abstract

About the bone marrow transplantation that high dose chemotherapy and total-body irradiation (TBI) are used for as conditioning regimen, a late toxicity may become the problem in the long-term survival patient. One of the toxicities which has been implied to be associated with TBI is secondary cacinogenesis. Between June 1995 and December 2010, 370 patients who were undergoing allogeneic hematopoietic stem cell transplantation using a TBI-based regimen at our department, were the subjects of this study. Eleven secondary cancers occurred in 10 patients. The median time from transplantation to diagnosis of a secondary cancer was 6.8 years. In this analysis, the cumulative incidence rate of secondary cancer at 5 and 10 years was 2.15% and 6.46%, respectively after TBI in our institution.

**Electronic supplementary material:**

The online version of this article (doi:10.1186/2193-1801-2-424) contains supplementary material, which is available to authorized users.

## Introduction

The conditioning regimen before allogeneic hematopoietic stem cell transplantation is intended to eradicate tumor cells and to promote immunosuppression to prevent graft rejection. A combination of cyclophosphamide and total body irradiation (TBI) is the most widely used regimen in transplantation for leukemia.

Patients who receive bone marrow transplantation underlie an increased risk for secondary cancers because of several risk factors, including radiation, chemotherapy, and immune stimulation. Several studies (Schneider et al. 2007; Bhatia et al. 1996; Witherspoon et al. 1989; Deeg & Witherspoon 1993; Witherspoon et al. 1992; Deeg et al. 1984) described the risk factors and incidence of secondary malignancy after transplantation.

We here report our single-center experience regarding second malignancy in patients treated with TBI-based regimen. This paper focuses on the occurrence of second solid cancer.

## Materials and methods

### Patients

Between June 1995 and December 2010, 370 patients who were undergoing allogeneic hematopoietic stem cell transplantation using a TBI-based regimen at our department, were the subjects of this study. Data were obtained from our bone marrow transplantation (BMT) database.

### Transplantation procedure

The first choice of the preparative regimen is cyclophosphamide (Endoxan) 60 mg/kg div day −3, -2 and full TBI 2 Gy x2/day day −6, -5, -4 for acute myeloblastic leukemia (AML), acute lymphoblastic leukemia (ALL), myelodysplastic syndrome (MDS), chronic myeloblastic leukemia (CML). Calcineurin inhibitor [Cyclosporine A (Sandimmun) 3 mg/kg/day, cdiv day-1~ or FK506 (Tacrolimus) 0.03 mg/kg/day, cdiv day-1~] plus Methotrexate (MTX) day 1, 3, 6, 11 were administered to prevent GVHD.

A purpose of use of the immunosuppressive drugs in pediatric stem cell transplant is to control GVHD not to become severe. About the duration of administration, we reduce and cancel the immunosuppressive drug when GVHD becomes the minor degree not to affect everyday life. The period until coming to become able to control is not fixed because there is individual difference. In other words, we continue using an immunosuppressive drug for years for the case that GVHD aggravates when dose reduction. The kind of the immunosuppressive drug uses cyclosporine in the case of transplant between blood relatives and tacrolimus in the case of between unrelated blood relatives interval like the transplant of the adult.

### Total body irradiation

Patients were treated in a mobile box made of 10 mm thick polymethyl methacrylate 600 mm wide by 2000 mm long by 400 mm high. The box is capable of moving up to 250 cm forward and backward on the rails with a constant speed. Beam intensity and moving velocity defined dose rate in TBI (Ban et al. 2001). Normally, beam opening of the linac is 400 cm × 10 cm. Leukemia patients were usually treated in the supine position for three fractions in the morning and in the prone position for three fractions in the evening. The center of the mobile box was selected to be a reference point to attain the prescribed dose. Beam intensity and moving velocity were determined based on the measurement of the doses in Mix-DP slab phantoms with an ionization chamber, but no corrections for patient body size were required due to the use of the mobile box. Dose rate was 150 MU/min in all cases. Most commonly, a pair of customized metal blocks was placed on the mobile box for lung shielding. The blocks were fabricated according to the lung shape, which was obtained by use of the X-ray film taken in the box. Lung shielding was performed in a fraction of TBI out of six fractions for three consecutive days in most cases.

### Statistical analysis

The probability of the incidence of secondary cancer was estimated using the Kaplan-Meier method.

### Patients

The patients were 236 males and 134 females. The median age at transplantation was 36 years old (range; 1–72). The median follow-up time for only survivors was 10.5 years (max; 16.4). A hundred thirteen patients (31%) received transplantation for acute AML, 117 patients (32%) for ALL, 39 patients (11%) for lymphoma, 34 patients (9%) for MDS, 41 patients (11%) for CML, and six patients (1.6%) for dyshematopoiesis. Two hundred forty patients (65%) survived at least 1 year after TBI.

The conditioning regimens included TBI with cyclophosphamide (CY) alone (72%), etoposide (VP-16) alone (10%), or a combination of CY and VP-16 (18%). For pediatric case, melphalan (L-PAM), antithymocyte globulin (ATG), thiotepa (TESPA), or fludarabine were administered for ten, one, one, and two patients, respectively. Graft-versus-host disease (GVHD) prophylaxis consisted in the majority of patients of cyclosporine-A associated to methotrexate, and FK506 associated to methotrexate in some patients.

## Results

### Secondary cancers

**Figure 1:**
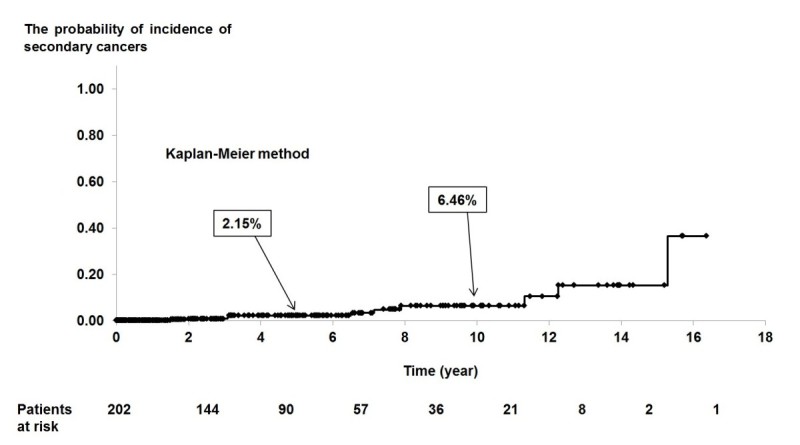
**The probability of incidence of secondary cancers after transplantation.**

Eleven secondary cancers occurred in 10 patients. One patient presented both esophageal and gastric cancer. The median time from transplantation to diagnosis of a secondary cancer was 6.8 years. The probability of incidence of secondary cancers at 5 and 10 years after transplantation was 2.15% (+/− 1.22%) and 6.46% (+/− 2.82%), respectively (Figure 1).

Secondary cancers were thyroid papillary carcinoma in one patient (7.8 years after TBI), sub-maxillary gland tumor in one patient (1.4 years), esophageal cancer in 2 patients (7.1 and 12.2 years), oral cavity carcinoma in 1 patient (15.2 years), gastric cancer in 2 patients (1.9 and 7.1 years), and ureteral cancer in 1 patient (6.4 years), border malignant ovarian tumor in 1 patient (11.3 years), extragonadal germ cell tumor in 1 patient (3 years), the head and neck cancer in 1 patient (3 years). Table 1 shows the clinical characteristics of the patients with a secondary cancer.

**Table 1:** **Clinical characteristics of the patients with a secondary cancer**

Secondary cancer	Sex	Age at TBI	Time to carcinogene sis (year)	Implant procedure	Primary tumor	State	Cause of death	Chronic GVHD	CTx regimen
Oral cavity carcinoma	F	17	15.2	allo-BMT	Lymphoma	Alive		-	ALL202-U
Esophageal cancer	F	39	12.2	allo-BMT	ALL	Alive		-	ALL202-O
Border malignant ovarian tumor	F	43	11.3	allo-BMT	ALL	Alive		+	ALL202-O
Thyroid papillary carcinoma	F	27	7.8	allo-PBSCT	Lymphoma	Alive		-	-
Both esophageal and gastric cancer	M	52	7.1	allo-BMT	MDS	Dead	Aspiration pneumonia	+	-
Head and neck cancer	M	55	3.0	allo-BMT	AML	Dead	Secondary cancer	+	IDR+AraC/HDAraC
Gastric cancer	F	17	1.9	allo-BMT	AML	Dead	Secondary cancer	+	IDR+AraC/HDAraC
Ureteral cancer	M	54	6.4	allo-BMT	MDS	Alive		+	-
Extragonadal germ cell tumor	M	48	3.0	allo-BMT	ALL	Dead	Secondary cancer	+	ALL202-O
Sub-maxillary gland tumor	F	29	1.4	allo-BMT	Lymphoma	Alive		+	-

*Abbreviation:TBI* total body irradiation; *CY* cyclophosphamide; *BMT* bone marrow transplantation; *PBSCT* peripheral blood stem cell transplantation; *ALL* acute lymphoblastic leukemia; *MDS* myelodysplastic syndrome; *AML* acute myeloblastic leukemia.

Among 10 patients with secondary cancer, six are alive at last follow-up. One patient with secondary gastric cancer had a recurrence of leukemia, and died on the primary disease 2.8 years after TBI. Three patients died from a reason due to secondary cancer.

## Discussion

This is a report about 10 patients with secondary malignancies after TBI. The study population includes 370 patients after undergoing TBI between 1995 and 2010 as a single center experience. Secondary solid cancers are seen after a latency period of 3 to 5 years after hematopoietic cell transplantation, subsequently, their incidence continues to rise with time. Several series (Schneider et al. 2007; Bhatia et al. 1996; Witherspoon et al. 1989; Deeg & Witherspoon 1993; Witherspoon et al. 1992; Deeg et al. 1984) have described the increased risk of secondary cancer after hematopoietic cell transplantation.

The Collaborative study between the CIBMTR and Fred Hutchinson Cancer Research Center (FHCRC) conducted a study among 19,229 recipients of allogeneic and syngeneic transplantation (Curtis et al. 1997). 72.8% of patients received TBI as the conditioning regimen. The cumulative incidence of secondary cancers at 5, 10, and 15 years after transplantation was 0.7%, 2.2% and 6.7%, respectively, compared to the general population rates of 0.3%, 0.6% and 0.8% (Curtis et al. 1997). In a similar report of the Late Effects Working Party in the European Cooperative Group for Blood and Marrow Transplantation, 1,036 consecutive patients surviving more than 5 years post transplants were recorded (Kolb et al. 1999). With a median follow-up of 10.7 years, the actuarial incidence of a solid tumor post-BMT was 3.5% +/− 0.6% at 10 years and 12.8% +/− 2.6% at 15 years and this incidence is 3.8-fold higher than that in an age-matched control population (*p* < 0.001) (Kolb et al. 1999). The University of Minnesota reported a series of 3,372 recipients of BMT (Baker et al. 2003). The majority of patients in this study (78%) received a regimen that contained radiation, delivered as a fractionated TBI (12.0 to 13.2 Gy) in most patients or as a single-fraction TBI (7.5 Gy), given in combination with cyclophosphamide or with other chemotherapy agents. After a median follow-up of 5 years 137 patients developed 147 second malignancies, compared with 4.3 expected from general population and the estimated actuarial incidence of any post-BMT malignancy was 9.9% +/− 2.3% at 13 years (Baker et al. 2003). The City of Hope National Medical Center reported 2,129 patients who had undergone BMT for hematologic malignancies (Bhatia et al. 2001). The conditioning regimens for patients with leukemia included TBI. The estimated cumulative probability for development of a solid cancer was 6.1% +/− 1.6% at 10 year which represents a two-fold increase in risk compared with general population (Bhatia et al. 2001). In this report, 11 solid secondary cancers occurred in 10 patients, and the cumulative incidence rate of secondary cancers at 5 and 10 years after transplantation was 2.2% and 6.5%, respectively, which is comparable with published studies evaluating the rate of secondary cancer after transplantation. According to the 2013 Annual Report of Nationwide Survey of HSCT by the Japan Society for HSCT, the incident probability of second cancer after CY+TBI and FL+TBI was 1.1% (CI: 0.7-1.6, N=1067) and 3.0% (CI: 2.2-4.1, N=509) at 3 years and 2.1% (CI: 1.4-3.3, N=198) and 5.2% (CI: 3.3-8.0, N=96) at 5 years after transplant, respectively.

In the pediatric experience reported by Socie et al. (2000), the Kaplan-Meier estimates of the probability of new invasive solid tumors at 5, 10, and 15 years after transplantation were 0.9% (+/− 0.6%), 4.3% (+/− 2.1%), and 11.0% (+/− 8.8%). Younger age at transplantation is a major risk factor of secondary solid cancers. Children less than 10 years of age also had a 33 to 36.6 fold higher risk of solid tumors than that expected in the general population. For Baker et al. (2003), children who had undergone transplantation when younger than 10 years had the highest risk (36.6 times as high as expected); the risk was 4.6 times as high as expected for those who were 10 to 29 year old at the time of transplantation and nearly normal for those who were 30 years or older (p <0.001). 86.5% of patients received TBI in the conditioning regimen. In this report, there was not the secondary solid carcinoma among 50 pediatric patients. It is cited in the reason that there are few numbers of people and that an observation period is short.

The risk factors for the development of post-transplant solid tumors included the use of radiation or the radiation dose in the conditioning regimen. TBI significantly increases the risk of second cancer especially if higher dose are delivered (Deeg & Witherspoon 1993). All patients with secondary cancer were performed TBI of 12 Gy in this report, but it is unknown whether a higher dose of TBI contributed to secondary cancer because almost all patients received 12 Gy.

Unusual cancers were frequently diagnosed as post transplantation secondary cancer. Cancers of the buccal cavity, liver, brain and central nervous system, thyroid, bone, connective tissue, salivary gland plus melanoma were significantly elevated compared to the general population for most authors (Curtis et al. 1997; Kolb et al. 1999; Baker et al. 2003; Bhatia et al. 2001). Although the risk of common adult cancer was little increased, TBI has been reported to increase the risk of breast cancer. In a cohort of 3,337 female 5-year survivors, the 25-year cumulative incidence of breast cancer was 17% in recipients of TBI compared to 3% in those who did not receive TBI as a part of their conditioning regime (Majhail 2008). In the results published by Socie et al. (2000), half the excess solid tumors in the youngest age group were cancers of the brain (observed cases, 9; expected cases, 0.22) or thyroid (observed cases, 4; expected cases, 0.02). In this report, a relative rare solid cancer like maxillary gland tumor or extragonadal germ cell tumor was seen and the carcinogenesis of secondary breast cancer and brain tumor was not observed. This fact will be a cause that we had few long-term observation cases.

From the epidemiologic data of atomic bomb survivors from Hiroshima and Nagasaki, radiation induced solid cancer is gradually increasing after 10 years from exposure. However, as shown in our and other reference research, it seems to be induced little earlier than atomic bomb survivor. We may have to follow the patients at least 10 years in order to focus our subject to the incidence of second solid malignancy. A larger number of irradiated patients with adequate longer follow-up periods are necessary to calculate a radiation carcinogenesis risk with reasonable accuracy.

Additionally, the definition of secondary cancer is too difficult. Some of cancer patients with surgery and without chemotherapy are suffered from another metachronous cancer. Although these cancers are so called secondary cancer or double cancer, they are not treatment-related cancer. The cause of treatment-not-related metachronous cancer may be related to hereditary and/or environment etc. From our research, some of patients seem to become secondary cancer very early from TBI compared with atomic bomb survivor or HD irradiated patients. In this study, we defined second cancer as the all new diagnosed cancers after TBI. Secondary primary cancer may also be induced by agents other than radiation; chemotherapeutic agents such as especially alkalylators, immunosuppressive agents, environmental exposures such as smoking and alcohol, hereditary disposition and so on.

We were not able to analyze statistically on the relationship between 11 secondary malignancies and TBI. It would be better to compare this study population to an age-matched control population, because age is a critical factor in determining radiation risk.

The number of enrolled patients (370 cases) may be substantially small for such an epidemiologic study. Many previously published reports involved several thousands of patients, such as Yokota’s report (2062 cases) (Yokota et al. 2012) and other reports (Curtis et al. 1997; Kolb et al. 1999; Baker et al. 2003; Bhatia et al. 2001; Socie et al. 2000; Majhail 2008).

## Conclusion

Various factors such as GVHD, high dose chemotherapy, or the use of CY have been nominated for risk factor of the secondary carcinoma other than TBI. The influence that TBI gives secondary cancer is hard to evaluate because a regimen including TBI is performed for all patients in this study. However, it is shown by the analysis of our institution that the risk of the secondary cancer rises by BMT including TBI just like the past reports and may not ignore the influence that TBI gives secondary cancer.

## Authors’ original submitted files for images

Below are the links to the authors’ original submitted files for images. Authors’ original file for figure 1
