# Supplementary figures and images for: Eleven secondary cancers after hematopoietic stem cell transplantation using a total body irradiation-based regimen in 370 consecutive pediatric and adult patients
Source: Springerplus. 2013 Aug 30;2(1):424. doi: 10.1186/2193-1801-2-424 (PMC3769541; doi:10.1186/2193-1801-2-424)

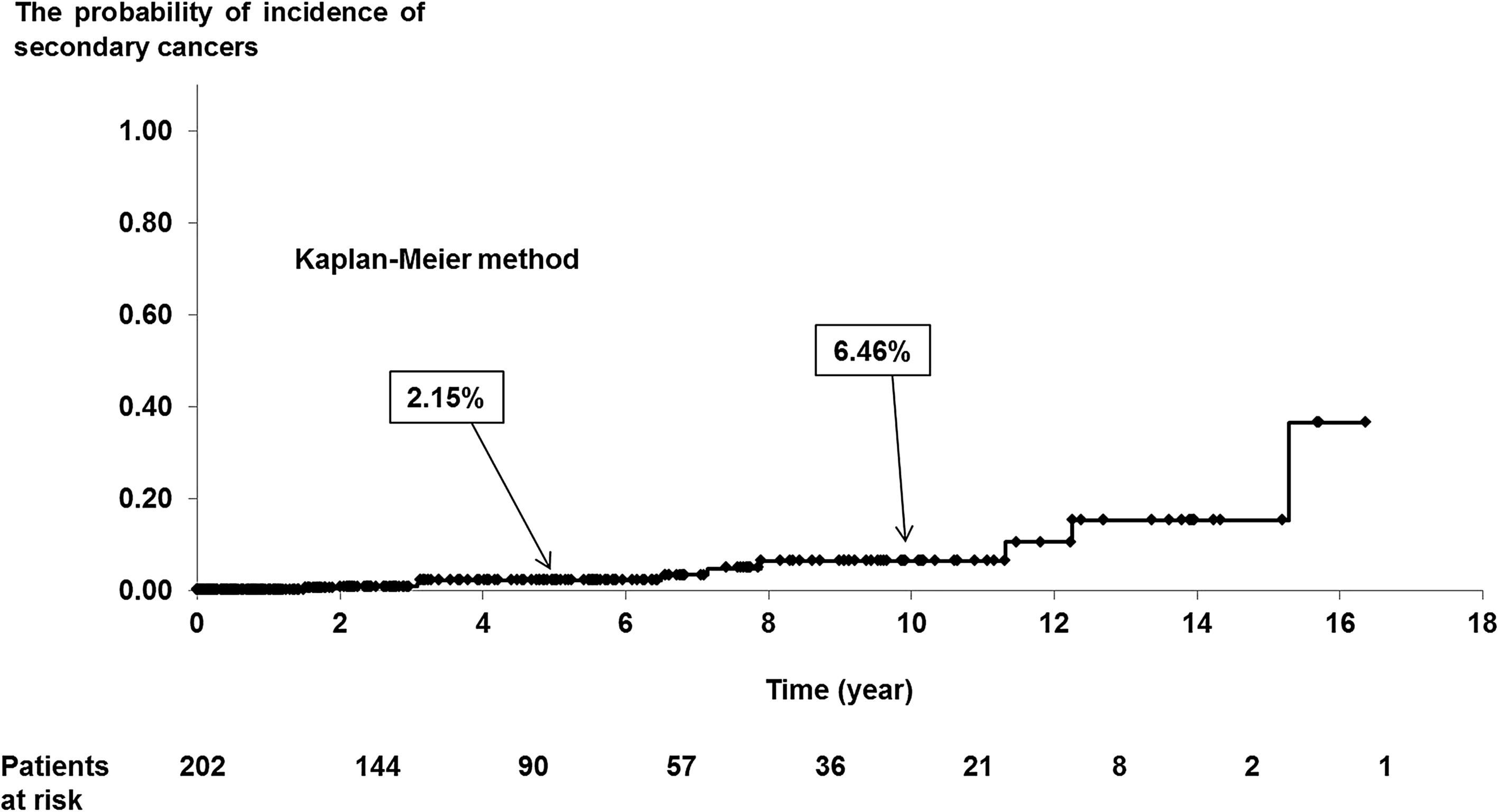

Supplement: Supplementary file 1 — Authors’ original file for figure 1 [file 40064_2013_498_MOESM1_ESM.tif]
